# A cluster randomized trial to evaluate the efficacy of a school-based behavioral intervention for health promotion among children aged 3 to 5

**DOI:** 10.1186/1471-2458-13-656

**Published:** 2013-07-15

**Authors:** José L Peñalvo, Gloria Santos-Beneit, Mercedes Sotos-Prieto, Ramona Martínez, Carla Rodríguez, Manuel Franco, Pedro López-Romero, Stuart Pocock, Juliana Redondo, Valentín Fuster

**Affiliations:** 1 Department of Epidemiology, Atherothrombosis and Imaging, Centro Nacional de Investigaciones Cardiovasculares (CNIC), Madrid, 28029, Spain; 2 International SHE Foundation, Barcelona, Spain; 3 Division of Epidemiology and Public Health, Universidad de Alcalá, Madrid, Spain; 4 Department of Epidemiology, Johns Hopkins Bloomberg School of Public Health, Baltimore, USA; 5 Department of Medical Statistics, London School of Hygiene and Tropical Medicine, London, UK; 6 Department of Cardiology, Mount Sinai School of Medicine, New York, NY, USA

**Keywords:** Comprehensive health, School-based intervention, Lifestyle, Behavior

## Abstract

**Background:**

The onset of inadequate behaviors leading to the development of risk factors for chronic diseases is known to occur early in life. An effective program for health promotion should therefore focus on children and their environment, as the starting point for behavior development. The overarching objective of the Program SI! (Salud Integral - Comprehensive Health) is to intervene at the school level, to establish and develop life-lasting habits that will help preserving health during adulthood. The Program SI! comprises five consecutive subprograms according to the five stages of education in Spain, the first being in preschoolers. This study aims to evaluate the efficacy of Program SI! to establish and improve lifestyle behaviors in children (preschoolers aged 3–5 years), their parents, and teachers, and also improving the school environment. A secondary objective is to evaluate improvements in cardiovascular health-related markers (anthropometric parameters, blood pressure, and dietary and physical activity patterns) in these same children.

**Methods/design:**

24 public schools from the city of Madrid (Spain) were allocated through stratified randomization to intervention or control. The intervention schools follow the Program SI!, which provides didactic units, emotions cards, healthy tips, and online resources. The intervention schools integrate the Program SI! into their scholar curriculum organized in four complete weeks during each academic year during the 3 years of preschool education. Control schools follow their normal curriculum. Primary outcomes are 1-year, and 3-year changes from baseline of scores for knowledge, attitudes, and habits (KAH) of children, their parents and teachers in regards to a healthy lifestyle. Secondary outcomes are 1-year, and 3-year changes from baseline in clinical and anthropometric parameters of children.

**Discussion:**

The Program SI! is a long-term health promotion program starting in 3 years old. It incorporates the traditional areas of intervention (diet and physical activity), introducing additional components such as knowledge of the human body and management of emotions to achieve a comprehensive intervention. The Program SI! is designed to be an effective, sustainable health promotion program for the adoption of healthy behaviors from early in life.

**Trial registration:**

Trial registration number: NCT01579708

## Background

Cardiovascular diseases (CVD) are the leading cause of death in the world [1]. It is now apparent that one of main determinants of CVD is the high prevalence of obesity and its comorbidities such as diabetes and hypertension [2]. Obesity also affects children, and it is especially relevant in south European countries [3-6]. The latest data from Spain showed a prevalence of 18.3% obese and 26.2% overweight children (7,659 children, 6–9 years old) [7].

Obesity is mostly a result of inadequate dietary habits and a sedentary lifestyle. It is well known that the development of adequate behaviors start at a very young age [8]. Effective health promotion initiatives directed to young children, should also involve their immediate environment, that is their parents [9,10], and their teachers, and use the school as the center of a multicomponent educational intervention [11-13]. Previous experiences of interventions developed within the school environment and aimed at preventing the development of risk factors have proven to be successful [14]. A recent review on school-based interventions concluded that the most effective programs are those that also include the family and focus on realistic, intermediate objectives such as changes in knowledge, attitudes, and lifestyle habits [15].

The Colombian Initiative for Healthy Heart Study [16], a multi-target educational intervention program based on Sesame Workshop materials aims to change children’s behavior through a comprehensive intervention [17]. It started on preschoolers (3 years old) and involved parents, teachers and the school as a whole. The study included intervention on three components (diet, physical activity, and body and heart) and was evaluated through assessing changes in three domains, knowledge, attitudes and habits (KAH) of children (1,216 children aged 3–5) after 6-months follow up of 14 schools enrolled on a school-based randomized intervention. The investigators found differences of low magnitude, though statistically of note in the knowledge, attitudes and habits (KAH) between the intervention and control groups [17]. Based on the Colombian Initiative, the Program SI! (Salud Integral = Comprehensive Health), was also designed to promoting cardiovascular health from early childhood with the acquisition of healthy habits that will set the basis for a healthier lifestyle during adulthood.

### Intervention program

The Program SI! entails four lifestyle-related components: 1) Diet, 2) Physical activity, 3) Knowledge of the human body and heart, and 4) Management of emotions. While the first two components are directly linked to a healthy lifestyle, the third component is necessary to understand the concept of health, and the fourth aims to prevent the development of behavioral disorders and drug abuse. The Program SI! is based on three domains of behavior development: 1) Knowledge, 2) Attitudes, and 3) Habits. The Program targets four population strata: 1) children, 2) parents, 3) teachers, and 4) the school environment. The Program SI! is implemented in the classroom on four complete weeks during the academic year during every school year (from 2011 to 2014). A summary of the Program SI! intervention including the minimum hours dedicated to each stratum are presented in Table 1. All four components are represented equally in all the activities and materials.

**Table 1:** Summary of the Program SI! Intervention components

**Strata**	**Objectives**	**Intervention activities**	**Intervention materials**	**Minimum hours**
**Children**	Acquiring KAH regarding the 4 components of Program SI!	- Classroom instruction	- Didactic units. Including 7 key activities per unit, and associated resources: Sesame Street audiovisuals, books and games, and cooking workshops, and tales on healthy living	70
		- Health Fair	- Sesame Street Emotion cards	
**Parents**	Improving KAH regarding the 4 components of Program SI!	- Program SI! by Dr. Fuster (video)	- Informative letters and leaflets	12
		- Health Fair	- Program SI! website	
			- Healthy tips	
**Teachers**	Improving KAH regarding to the 4 components of the PSI!	- Program SI! by Dr. Fuster (video)	- Training booklet, and associated audiovisual material	30
		- Program SI! capacity building:	- Intranet continuing education tools	
		intensive training for teachers, and SHE Foundation’s liaisons	- Program SI! website	
		- Continuing counseling from SHE Foundation’s staff to the liaisons		
		- Health Fair		
**School**	Improving the school environment in regards of healthy environment	- Program SI! by Dr. Fuster (video)	- Document of healthy recommendations in schools	20
		- Periodical meetings between principals and SHE Foundation’s liaison	- Program SI! website	
		- Health Fair		

*KAH*, Knowledge, attitudes and habits.

### Aims

The primary aim is to evaluate the efficacy of the Program SI! for preschoolers (3–5 years old) to change the knowledge, attitudes, and habits (KAH) of children, their parents, and teachers in regards to a lifestyle (diet, physical activity, knowledge of the human body, and emotion management) after 3 years of a school-based intervention. The secondary aim is to evaluate the efficacy of the Program SI! for preschoolers to improve cardiovascular health-related risk markers in children, namely anthropometric parameters (weight, height, waist circumference, skinfold thicknesses), and blood pressure after 3 years of intervention. We followed the updated CONSORT Statement for cluster randomized trials [18] to avoid bias in the design and therefore in the reporting results.

## Methods/design

### Study design

The evaluation of the Program SI! in children aged 3 to 5 years (and their parents, teachers, and school environment) was designed as a cluster-randomized controlled open label intervention in the city of Madrid. The unit of randomization, intervention, and analysis is the participating school.

### Sample size calculation

**Figure 1:**
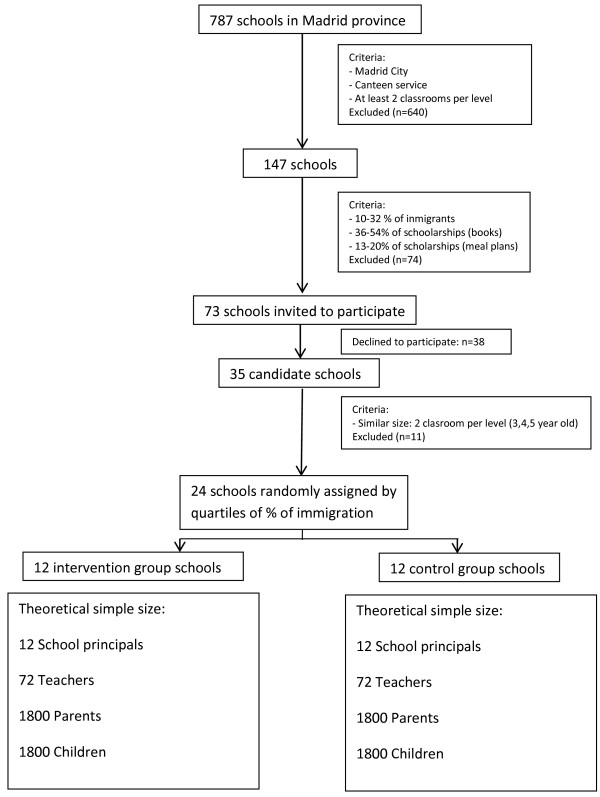
Flow diagram of school selection and allocation to intervention.

In previous school-based interventions, intra-class correlation (school-group effect) coefficients ranged from 0.05-0.30 for scores that measured academic results [9,19,20]. Taking this range into consideration, and using an estimated intra-class correlation of 0.15, we calculated 20 schools (50 students per level) as sufficient to detect differences of 0.8 in overall scores with a power greater than 80%, and type-I error 0.05 [21]. In anticipation of possible drop outs during the study, a total of 24 schools were randomized, 12 in each group. According to this design, the theoretical sample size for each stratum is presented in Figure 1.

### Selection of schools and allocation to intervention

During the academic year 2010/11, the number of public schools in Madrid area was 787. In order to homogenize our sample, a series of inclusion criteria were applied to obtain a final subsample with comparable socio-economic characteristics (Figure 1). The study focused only on schools located in the city of Madrid, having full canteen services, and with two or more classrooms per preschool level (needed to attain the 50 students per level used in sample size calculation). This comprised a total of 174 schools. We further gathered data on % immigration and % scholarships, and excluded those schools on the extremes of the distribution. A total of 73 remaining schools were invited to participate on a 1-day meeting where the fundamentals of the Program SI! were presented. A total of 35 schools agreed to participate, from which the final 24 were chosen, excluding larger schools with more than 2 classrooms per level in order to have a similar number of students in each school.

### Randomization process

This trial is based on a hierarchical design, where schools are the experimental units that receive the intervention and therefore are the units that are either randomized to intervention or control group. We randomized the schools using percentage of immigration as stratification factor with four levels (according to quartiles) to ensure that the groups were balanced by differences in cultural background.

### Data collection

#### Primary variables

To assess the impact of the Program SI!, children, parents and teachers participating in the study are evaluated before and after the intervention using an adapted version of the questionnaires developed in the Colombia Initiative for Health Heart Study. These include questions regarding KAH in relation to diet, physical activity and knowledge of the heart and body [17,22]. To further explore the possible changes in the dietary patterns of the children and parents, we used questionnaires previously developed in the studies enKid [23] and Predimed [24] measuring adherence to the Mediterranean diet. We also evaluate possible changes in the physical activity of the children through a physical activity test that was also used in the enKid study [23]. For the evaluation of the fourth component of the program (management of emotions) we use the Test of Emotional Comprehension (TEC) [25]. The number of items of each questionnaire and the assessed component are summarized in Table 2. Finally, to assess the impact of the Program on the school environment, a questionnaire is given to the school’s Principal corresponding to the involvement of the center on Program-related activities. All the evaluation through questionnaires was carried out by trained psychologists.

**Table 2:** Distribution of questionnaires (including number of items) by components, domains, and strata

	**Teachers**	**Parents**	**Children**
**Diet**	KAH (13)	KAH (12)	KAH (10)
		H (14)	H (16)
**Physical activity**	KAH (12)	KAH (11)	KAH (6)
			H (2)
**Human body**	KA (5)	KA (5)	KA (5)
**Emotions**	-	-	TEC (26)

#### Secondary variables

All children participating in the study are evaluated at baseline and 1- and 3-years thereafter. Anthropometric data were obtained according to the guidelines of the International Society for the Advancement of kinanthropometry (ISAK). All examinations were performed by trained examiners in small groups of children. Body weight measurements were taken with a Model 803, Seca® scale (Hamburg, Germany). Mobile stadiometers (model 213, Seca®) were used to measure body height. To establish the cut-offs of obesity we followed the criteria by Cole and co-workers [26,27], therefore different categories were created: thinness grade 3, thinness grade 2, thinness grade 1, normoweight, overweight and obesity. Waist circumference was measured halfway between the lower costal border and the iliac crest using a flexible, nonelastic Holtain tape (Crymych, UK), skinfold thicknesses (triceps and subscapular) are measured using a calibrated Holtain caliper on the right hand side of the body. Percentage of body fat was calculated based on Slaughter equations [28]. Blood pressure was measured twice, with a 2–3 min interval between measurements with Omron Small arm cuff (arm circumference 17–22 cm adapted for children, model M6, Omron® CS2). The child is seated in relaxing conditions with the right arm semi-flexed at heart level. Normal blood pressure and hypertension is defined according to the criteria of the Fourth Report on the Diagnosis, Evaluation, and Treatment of High Blood Pressure in Children and Adolescents [29].

### Outcomes

Outcome variables of the study for comparing intervention versus control are divided into primary and secondary, depending on the target objective.

#### Primary outcomes

–Changes from baseline to 1 year and 3 years in the scores of:

○ KAH questionnaires of the children with respect to dietary habits, physical activity, and human body.

○ TEC questionnaire assessing emotion’s management in children.

○ KAH questionnaires of the parents and teachers regarding a healthy lifestyle.

–Changes from baseline to 1 year and 3 years in the overall score for teachers, parents and children.

–Changes from baseline to 1 year and 3 years in the school towards healthier environment based on the recommendations form Program SI!

#### Secondary outcomes

–Changes from baseline to 1 year and 3 years in anthropometric parameters (weight, height, waist circumference and skinfold thickness) and blood pressure in children.

### Statistical methods

For each of the four components of the study, we estimate the difference between the intervention and control groups from the individual evaluation scores based on the questionnaires used in each component. All of the statistical analysis and algorithms of the samples will be performed using the STATA, version 12.0 (STATACORP, College Station, Texas, USA). The differences between the control and intervention groups statistical significance will be obtained using mixed linear models. This methodology allows us to account for the hierarchical cluster randomized design of the study, and to adjust for baseline variables and the effect of clustering. The dependent variables analyzed will be the defined scores for each of the 4 components.

### Ethical concerns

The study has the approval of the Regional Committee for Clinical Research Ethics (CEIC-R) of Madrid Area. Informed written consent to participation is required from the parents or legal guardians of the children to become part of the study, both in the intervention and control group schools, which shall specify the purpose of the study and the intervention, as well as various tests and measures that will be taken. Once the parents have signed the informed consent, they and their child or children will be formally included in the evaluation study. Informed written consent to participation from the teachers should also be signed. We assure the confidentiality of all of the data submitted by the participants. The information collected is treated according to the Organic Law 15/1999 for the Protection of Personal Data.

## Discussion

The Program SI! is introduced in the school as early as in preschool level, since research has shown that development of adequate behaviors starts at a very young age [8]. The Program SI! is one of the largest health promotion program starting in preschoolers (3 to 5 years old) in Europe. It incorporates the traditional components of lifestyle behavior (diet, and physical activity) plus newer components: knowledge of the human body and management of emotions that also impact greatly on health promotion. Effective school-based interventions need to be long term and sustainable. One of the advantages of the Program SI! is that it is integrated into the school’s own curriculum, thus not becoming an extra burden for the school. The Program SI! also supports the teachers with easy access to materials and information. This is of importance due to the critical role that teachers play as key agents for change in children’s health behaviors. We have successfully developed methods to evaluate the efficacy of the Program SI! with input from the teachers, parents and children that will back up the great implications of the Program on school-based health promotion policy and practice, not only in Spain but also it could be extended to other European countries as a tool for behavior change early in life.

## Abbreviations

KAH: Knowledge, attitudes and habits; TEC: Test for emotional comprehension.

## Competing interests

The authors declared that they have no competing interest.

## Authors’ contributions

JLP coordinated and designed the study, and drafted the manuscript. GSB, and MSP, assisted with data collection, participated in the study design, and helped drafting the manuscript. RM, CR, MF, and JR contributed to the design, and implementation of the study. PLR and SP, participated in the design of the statistical analysis. VF, conceived of the study and helped drafting the manuscript. All authors read and approved the final manuscript.

## Pre-publication history

The pre-publication history for this paper can be accessed here:

http://www.biomedcentral.com/1471-2458/13/656/prepub
